# Neural Coding of Vibration Intensity

**DOI:** 10.3389/fnins.2021.682113

**Published:** 2021-11-11

**Authors:** Wanjoo Park, Sung-Phil Kim, Mohamad Eid

**Affiliations:** ^1^Engineering Division, New York University Abu Dhabi, Abu Dhabi, United Arab Emirates; ^2^Department of Biomedical Engineering, Ulsan National Institute of Science and Technology, Ulsan, South Korea

**Keywords:** haptics, neural signal processing, vibration, sensation, alpha ERD

## Abstract

Vibrotactile feedback technology has become widely used in human–computer interaction due to its low cost, wearability, and expressiveness. Although neuroimaging studies have investigated neural processes associated with different types of vibrotactile feedback, encoding vibration intensity in the brain remains largely unknown. The aim of this study is to investigate neural processes associated with vibration intensity using electroencephalography. Twenty-nine healthy participants (aged 18–40 years, nine females) experienced vibrotactile feedback at the distal phalanx of the left index finger with three vibration intensity conditions: no vibration, low-intensity vibration (1.56 g), and high-intensity vibration (2.26 g). The alpha and beta band event-related desynchronization (ERD) as well as P2 and P3 event-related potential components for each of the three vibration intensity conditions are obtained. Results demonstrate that the ERD in the alpha band in the contralateral somatosensory and motor cortex areas is significantly associated with the vibration intensity. The average power spectral density (PSD) of the peak period of the ERD (400–600 ms) is significantly stronger for the high- and low-vibration intensity conditions compared to the no vibration condition. Furthermore, the average PSD of the ERD rebound (700–2,000 ms) is significantly maintained for the high-vibration intensity compared to low-intensity and no vibration conditions. Beta ERD signals the presence of vibration. These findings inform the development of quantitative measurements for vibration intensities based on neural signals.

## 1. Introduction

Humans are surrounded by vibrations that are extremely important to how the ambient environment is perceived. In human–computer interaction applications, vibration feedback technologies seem to gain more popularity, compared to force feedback counterparts, due to their low-cost, wearability, and expressiveness (Chouvardas et al., 2008). With the widespread use of wearable devices with vibration capabilities, understanding how humans perceive vibrations is essential for the design of vibrotactile interfaces. For instance, understanding how the physical properties of vibration such as intensity, duration, and frequency influence vibration perception is crucial for the design of effective vibration-mediated interfaces.

Human perception of vibrotactile signals has been the subject of several psychophysical studies that are based on self-reporting and behavioral assessment (Verrillo et al., 1969). Many aspects of vibration perception have been studied in great detail, including detection threshold (Reynolds et al., 1977; Moshourab et al., 2016), perception of vibration intensity and equal sensation curve (Giacomin et al., 2004; Mansfield and Maeda, 2005), frequency discrimination (Tommerdahl et al., 2005; Mahns et al., 2006), emotional responses (Reynolds et al., 1977; Réhman, 2010), among others. Most previous studies used self-reporting and/or behavior analysis in order to evaluate the user experience. An emerging approach to measure the mental experience of vibration is to utilize brain imaging technologies such as electroencephalography (EEG) or functional magnetic resonance imaging (fMRI) in order to provide quantitative, real-time, and non-intrusive evaluation of vibration experience (Coghill et al., 1994; Harrington and Downs III, 2001; Simons et al., 2005; Kim et al., 2016). Since neuronal information processing for vibration occurs at a millisecond timescale (Mackevicius et al., 2012), EEG plays an important role in assessing vibration information processing due to its high temporal resolution (Burle et al., 2015).

Several EEG analytical methods are utilized for the quantitative exploration of vibration, including time domain analysis such as event-related potentials (ERP) (Ryun et al., 2017), frequency domain analysis such as power spectral density (Khasnobish et al., 2018) or steady-state evoked potential (SSEP) (Timora and Budd, 2013; Moungou et al., 2016), time-frequency analysis such as event-related desynchronization/synchronization (ERD/ERS) (Choi et al., 2017), and functional connectivity such as phase locking value (PLV) (Hari, 1980; Langdon et al., 2011). Previous EEG studies examined how the human brain represents various vibrotactile properties, most notably the frequency and intensity of vibration. Early studies demonstrated that vibrotactile frequency information are encoded in the P50 and P100 components of the ERP waveform in the postcentral gyrus of the primary somatosensory cortex and the parietal operculum of the secondary somatosensory cortex (Hämäläinen et al., 1990). A recent study examined the neural correlates of vibration intensity by considering three vibration intensities (0.25, 0.38, and 1.3 g) (Choi et al., 2020). Results demonstrated that the maximum and minimum peak, and peak to peak values of somatosensory evoked potential (SEP) patterns in the C3 somatosensory area increased as the stimulus intensity increased.

ERD, a localized power attenuation in the EEG rhythm, is associated with increased activation of the somatosensory and motor cortices during sensorimotor processing (Neuper et al., 2006). In particular, alpha (8–12 Hz) and beta (18–30 Hz) ERD oscillations are strongly associated with tactile sensation (Buchholz et al., 2014). On the other hand, localized power attenuation ERP is known to play a significant role in coding tactile perception (Tang et al., 2020). Two components of the ERP waveform will be considered: P200 and P300. P200 or P2 component, a positive deflection peaking around 100–250 ms after the stimulus, may reflect the sensation-seeking behavior of an individual (e.g., detection of vibration; Sur and Sinha, 2009). On the other hand, P3 component is associated with cognitive functions such as the identification of the vibration intensity (Sur and Sinha, 2009). Therefore, the P2 and P3 components of the ERP waveform as well as the alpha and beta band oscillations will be considered in this study.

The aim of this study is to systematically examine brain correlates associated with the intensity of vibration when applied at the distal phalanx of the left index finger. We hypothesize that the vibration intensity modulates P2 and P3 components as well as alpha and beta band oscillations. Three levels of vibration intensities are applied: no vibration, low-intensity vibration (1.56 g), and high-intensity vibration (2.26 g). This study contributes to developing quantitative measure of vibration intensity perception, and informs both the cognitive mechanisms associated with vibration intensity perception and the development of future vibration-enabled interfaces.

## 2. Materials and Methods

### 2.1. Participants

Twenty-nine healthy volunteers (nine females) participated in the experiment. The inclusion criteria were an age range of 18–55 years, right-handedness, and normal or corrected-to-normal vision/hearing. The exclusion criteria were a person with orthopedic hand conditions or with a history of neurological or psychiatric disease. All participants were students from Ulsan National Institute of Science and Technology recruited by an online call for participation. Thirteen participants are aged between 18 and 25 years old, 14 participants are aged between 25 and 30 years old, and two participants are aged between 30 and 40 years old. Eleven participants had previous experience using a haptic device. All participants were informed about the purpose of the experiment, and written informed consent was obtained prior to participation. The study was carried out with an approved protocol by Institutional Review Boards of New York University Abu Dhabi and Ulsan National Institute of Science and Technology (HRPP–2020–80). The experiment was conducted under the guidelines for prevention of novel coronavirus infection, Ulsan National Institute of Science and Technology, Republic of Korea.

### 2.2. Experimental Setup

**Figure 1:**
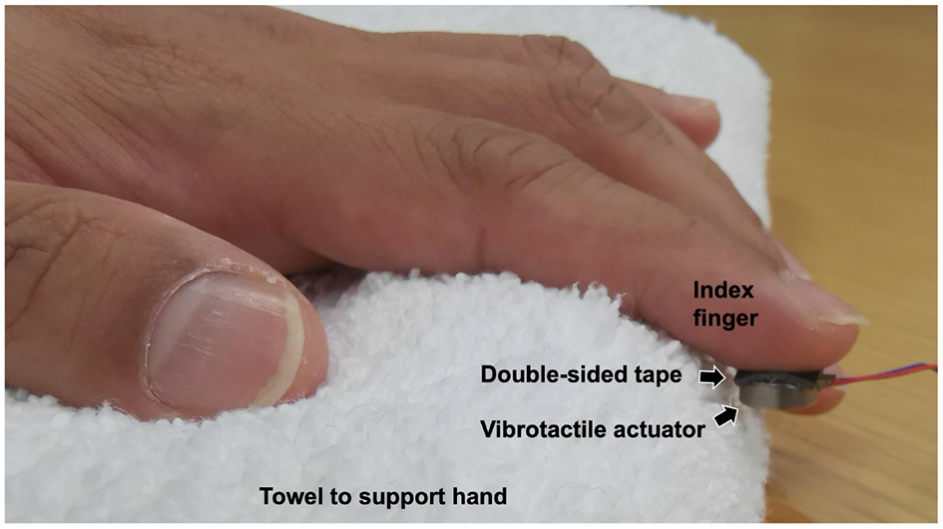
Participant's hand and a vibrotactile actuator.

A Pico Vibe 310–177 (Precision Microdrives) vibrotactile actuator was used to provide different levels of vibration intensity to the participants. The actuator has a 10 mm diameter and a 3.4 mm thickness. Figure 1 shows how the vibrotactile actuator was attached to the participant's left index finger. The participant's finger was wiped with an alcohol swab before the experiment. The vibrotactile actuator was attached to the participant's left index finger using a double-sided tape (3 M, model 5925), cut in a size of 10 × 10 [mm] (0.64 mm thickness). The left hand was supported with a towel to minimize hand movement during the experiment (as shown in Figure 1). The vibration motor was properly attached to the finger with no contact with the towel so the vibration stimulation is not attenuated.

Three levels of vibration intensity were used for the experiment; no vibration, low-intensity vibration, and high-intensity vibration. The stimulation intensities were controlled by adjusting the duty cycle of the pulse width modulation (PWM) signal of the Arduino microcontroller that controls the vibrotactile actuator. Increasing the duty cycle of the PWM signal increases the effective voltage applied to the actuator and thus the vibration intensity. The high and low intensity of vibration were measured using the optoNCDT 1750 vibrometer to be 2.26 and 1.56 g, respectively. These vibration intensity levels are perceptually distinguishable as confirmed through a pilot study. The frequency of the high and low vibration was in the range of 200–240 Hz, which is extremely difficult to distinguish for humans (Merchel and Altinsoy, 2020).

The stimulation software was developed using Presentation (a software by Neurobehavioral Systems, Albany, CA, USA). This software controls visual and auditory cues and synchronizes these cues with the vibrotactile stimulation through an Arduino microcontroller, as well as records event triggers in the EEG system. Neurological activities during the experiment were recorded with 1,000 Hz sampling rate using a 32-channel EEG device and amplified in the EEG recording system (BrainAmp by Brain Products, Munich, Germany). TP9 and TP10 electrodes were used for ground and reference channel, respectively. The experiment data are publicly available at: https://osf.io/j9s2q/.

### 2.3. Procedure and Evaluation Metrics

Before starting the experiment, participants completed a training session to get acquainted with the experimental setup and protocol. Participants were then asked to sit comfortably in order to minimize movements during the experiment.

**Figure 2:**
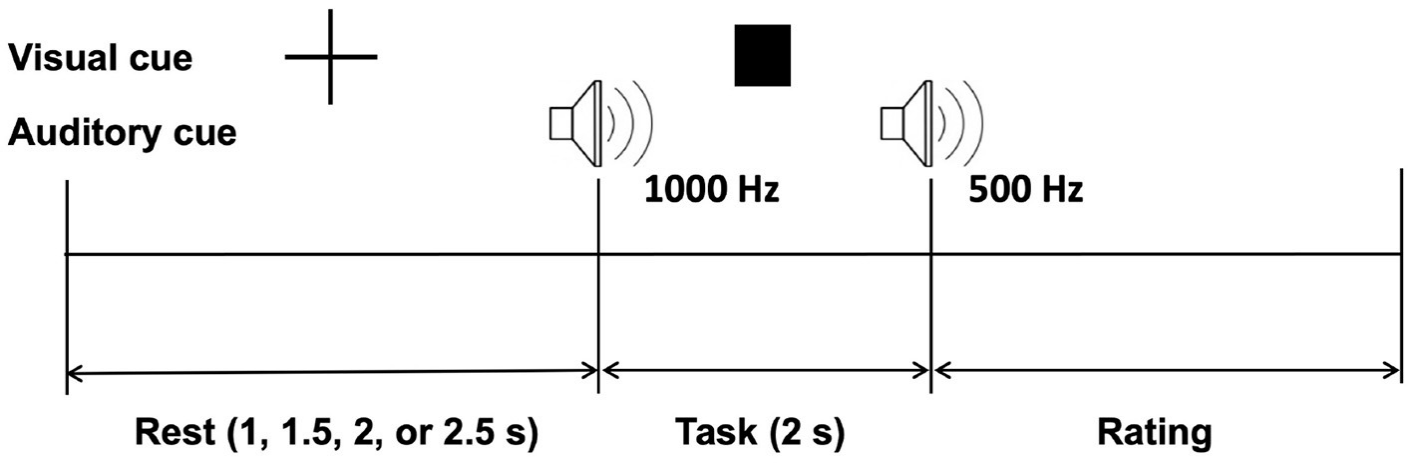
Experimental paradigm.

Figure 2 shows the experimental protocol. One trial consisted of rest, task, and rating periods. The rest period was randomly set to 1, 1.5, 2, or 2.5 s to prevent participants from predicting task cues. A visual fixation was displayed during the rest period to draw the user's attention to the assigned task. A square-shaped visual cue and a 1,000 Hz beep auditory cue announced the start of the task. Participants were instructed not to move their finger and just feel the vibration at the tip of their left index finger. The three levels of vibration intensities were displayed in this task period with random sequence to avoid any short-term memory or learning effects. A beep sound of 500 Hz indicated the end of the task. Immediately after the square-shaped visual cue disappeared, participants were asked to rate their experience using a 5-ratings scale (1, “I didn't feel any vibration”; 2, “I felt a very weak vibration”; 3, “I felt a weak vibration”; 4, “I felt a strong vibration”; and 5, “I felt a very strong vibration”). The user provided the rating input with the right hand via a numeric keypad (one to five number key). It is worth noting that the study involves multimodal stimulation, however visual and auditory stimuli were the same in all three vibration intensity conditions.

During the experiment, one trial took 3–6 s depending on the rest time and the self-reporting response time. One run consisted of 30 trials (10 trials for each vibration intensity level), for a total of about 2.5 min for one run. All participants completed ten runs separated by short breaks to reduce fatigue. A total of 100 trial data were recorded for each vibration intensity level per participant. For behavioral analysis, we investigated how participants rated each stimulus. This was to check if participants were able to clearly distinguish the three levels of vibration intensity.

For the preprocessing of the EEG data, the EEGLAB toolbox was utilized (Delorme and Makeig, 2004). EEG analysis was divided into time course power spectral density (PSD) and ERP analysis. For the time course PSD analysis, two EEG data streams corresponding to the outside locations (FT9 and FT10) were removed. Band pass filters with different frequency ranges were used. A zero-phase finite impulse response filter with a Hamming window was used for band pass filtering (0.1–55 Hz). The artifact subspace reconstruction method was applied to remove eye movement and muscle artifacts. Then, EEG signals were re-referenced using the common average reference (Binnie et al., 2003). The filtered EEG signal was divided into epochs (−1,000 to 2,000 ms) corresponding to the three vibration intensity levels and 1,000 ms before the onset was used as the baseline. After preprocessing, power spectral density of alpha (8–12 Hz) and beta (13–30 Hz) bands at each channel were computed via short-time Fourier transform with a 500 ms Hamming window, sliding by 50 ms.

The differences associated with the three vibration intensity levels (no vibration, low-intensity vibration, and high-intensity vibration) were analyzed through the topography of alpha and beta frequency bands in order to find areas of the brain that are associated with the vibration intensity. The contralateral and ipsilateral motor and somatosensory areas were the regions of interest. Time course alpha/beta PSDs during the task period in the bilateral somatosensory and motor cortices were investigated. The average PSD values for each vibration intensity level for all participants within the selected regions were compared. Box plots were used to show data distribution. On each box, the central mark indicates the median. The bottom and top edges of the box indicate the 25th (Q1) and 75th (Q3) percentiles, respectively. The whiskers extend to the most extreme data points not considered outliers. The outliers are defined as being any point of data that lies over Q3 + 1.5 × (Q3–Q1) or below Q1–1.5 × (Q3–Q1) and outliers are plotted individually using the “+” symbol. All data points including outliers were used for statistical analysis. One-way analysis of variance (ANOVA) or Kruskal–Wallis tests were used depending on whether the data followed a normal distribution by Jarque–Bera test. Then, the Holm–Bonferroni correction was used as a *post-hoc* to counteract the problem of multiple comparisons.

For the ERP analysis, a zero-phase finite impulse response filter with a Hamming window was used for band pass filtering (1–30 Hz). The artifact subspace reconstruction method was applied to remove eye movement and muscle artifacts (Mullen et al., 2013). Then, EEG signals were re-referenced using mastoids, T7 and T8. The filtered EEG signal was divided into epochs (−100 to 500 ms) corresponding to the three vibration intensity levels. The average of the EEG signals for the three vibration intensity conditions for each participant for 100 ms before the stimulation onset was used as the baseline. The time window for the ERP waveform is examined in the range of 150–275 ms for P2 (Correll et al., 2006) and 250–500 ms for P3 (Polich, 2007). The Jarque–Bera test was used to verify if the data followed normal distribution and box plots were used to show data distribution. All data points including outliers were used for statistical analysis. One-way ANOVA or Kruskal–Wallis tests were used depending on whether the data followed a normal distribution, and the Holm–Bonferroni correction was used as a *post hoc* to counteract the problem of multiple comparisons. Three areas of the brain were considered for the ERP analysis, namely the middle frontal (Fz), middle central (Cz), and middle parietal (Pz) channels.

## 3. Results

### 3.1. Behavioral Analysis

**Figure 3:**
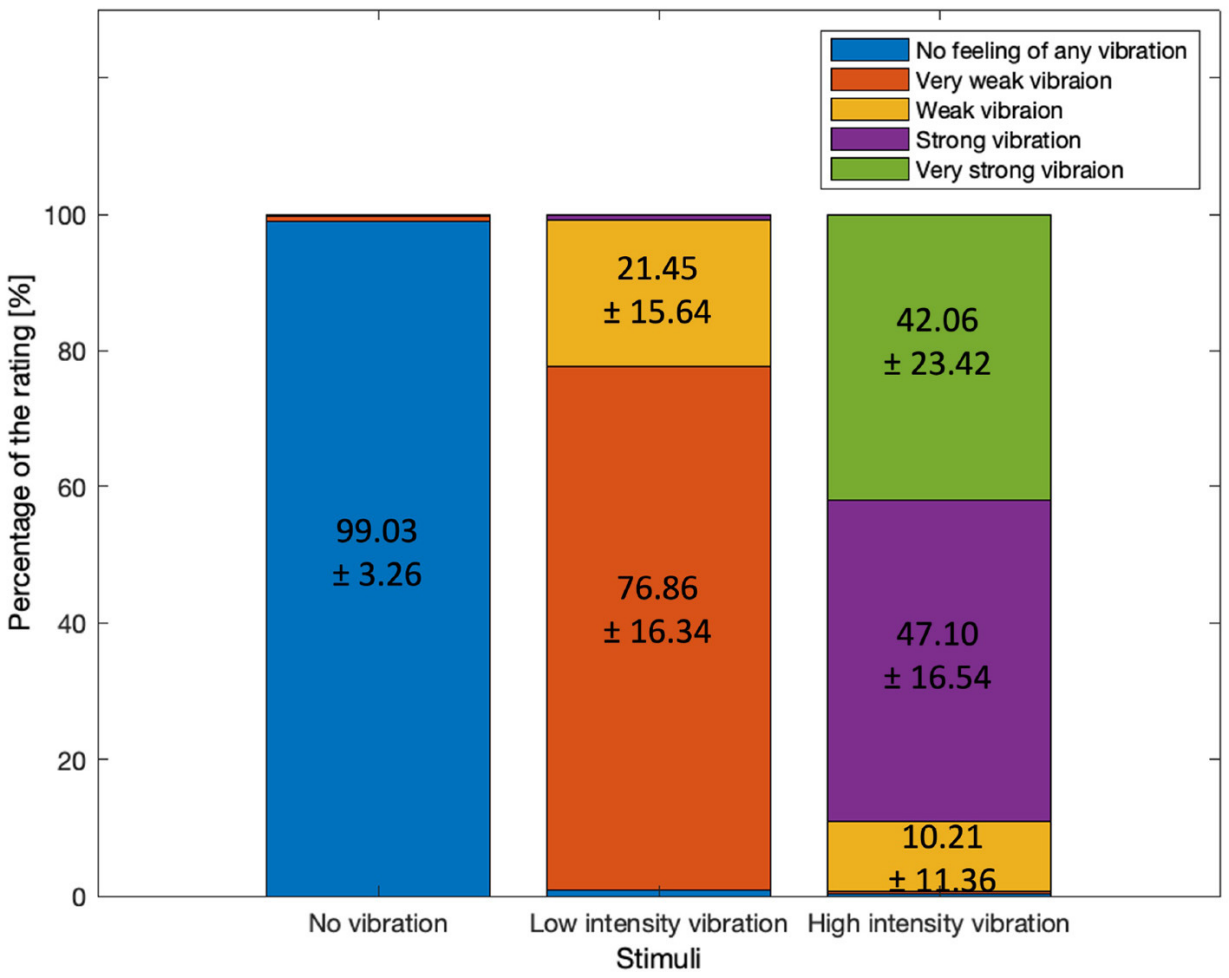
The percentages of the rating by three different stimuli after the task period. Mean ± standard deviation among the participants.

Participants' ratings for the three vibration intensity levels were examined (shown in Figure 3). For the no vibration condition, 99.03±3.26 percentage of the responses confirmed that no vibration was felt. For the low-intensity vibration, 76.86±16.34 percentage of respondents perceived the stimulus as very weak vibration while 21±15.64 percentage perceived it as weak vibration. In total, 98.31 percentage of the participants reported very weak or weak vibration for the weak vibration level. This confirmed that the no vibration and low-intensity vibration conditions were clearly perceived as expected. However, the perception of the high-intensity vibration was less consistent; 10.21±11.36 percentage of the responses were weak vibration, 47.10±16.54 percentage were strong vibration, and 42.06±23.42 percentage were very strong vibration. Unexpectedly, more than 10 percentage of the participants rated high-intensity vibration as weak. In addition, the standard deviation among the participants in case of the strong vibration stimulus was very large, 23.42. Unlike no vibration and low-intensity vibration, high-intensity vibration was rated differently (weak, strong, or very strong) depending on personal standards. It is assumed that the participants' personal experience and standards for the vibrotactile stimulus being strong or very strong were different. Since the individual standards for vibration intensity are different, it is expected that it would be meaningful to investigate the differences in EEG according to the perceived intensity, however it was difficult to compare due to the limited sample size.

### 3.2. Power Spectral Density Analysis

**Figure 4:**
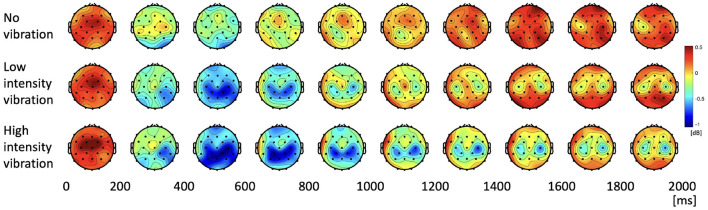
Topographies of alpha power spectral density during the task period.

We investigated how the alpha and beta frequency bands varied according to the three vibration intensity conditions over the two-second task. Figure 4 shows the power topographies of the alpha frequency band during the task period. In the first 200 ms of the task, alpha power increased for all the vibration intensity conditions. After that, in the case of low- and high-vibration intensity conditions, event-related desynchronization in the contralateral somatosensory and motor cortices was observed up to 400 ms. After 400 ms, the alpha ERD appeared bilaterally in the low- and high-intensity vibration conditions, which rebounded again after 800 ms in the case of low-intensity vibration. However, in the case of high-intensity vibration, alpha ERD was sustained until the end of the task period.

**Figure 5:**
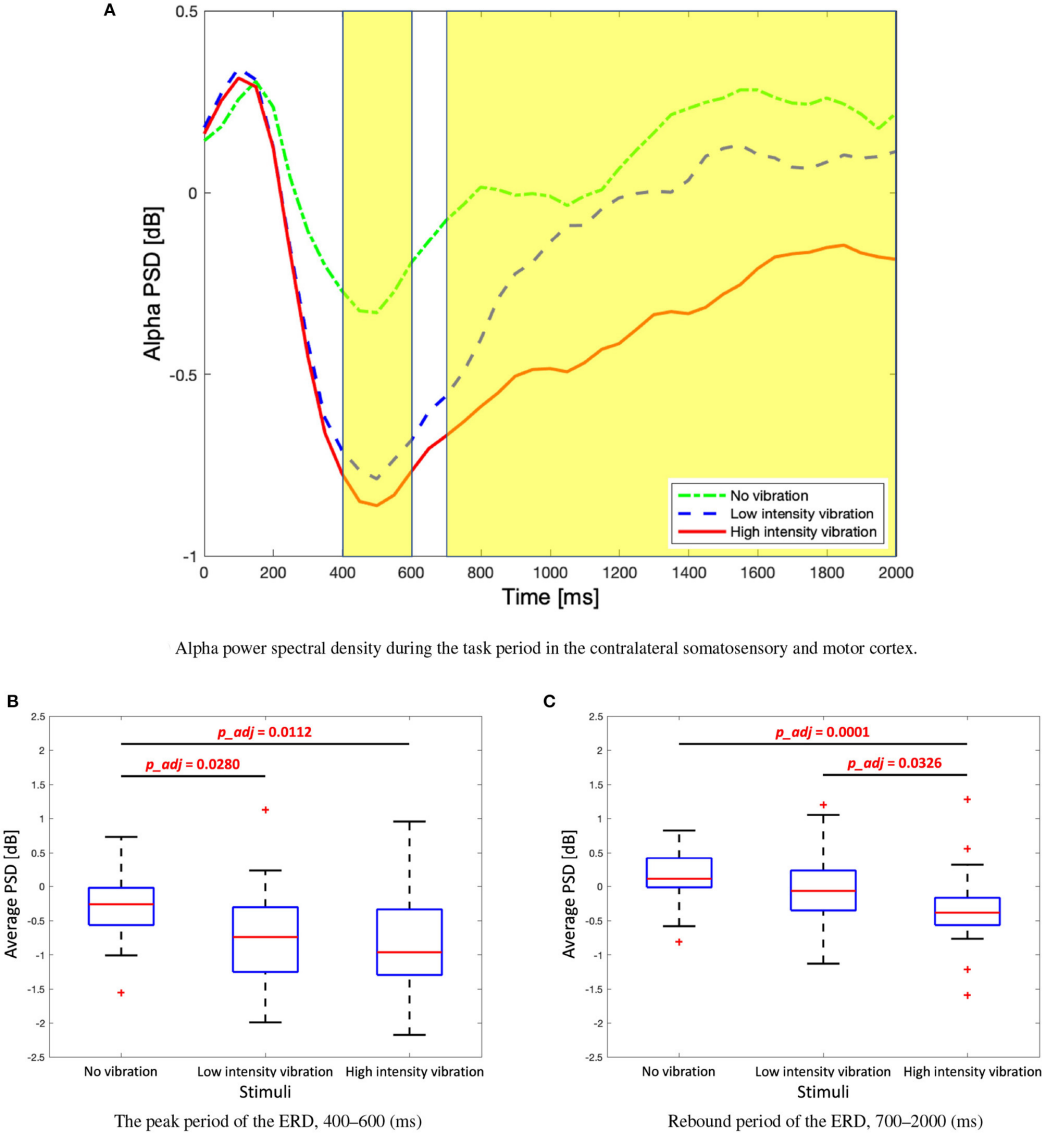
**(A)** Time course alpha power spectral density during the task period in the contralateral somatosensory and motor cortex (C4, Cp2, and Cp6). Two highlighted periods indicate significant differences among three stimuli. **(B)** Box plots to show average alpha power spectral density of the first highlighted region, the peak period of the event-related desynchronization (ERD). One-way analysis of variance (ANOVA), *F*_(2, 86)_ = 5.76, Holm–Bonferroni correction. **(C)** Box plots to show average alpha power spectral density of the second highlighted regions, rebound period of the ERD. Kruskal–Wallis test, *H*_(2)_ = 19.02, Holm–Bonferroni correction.

In order to observe the changes in the alpha band in the contralateral somatosensory and motor cortices (C4, Cp2, and Cp6) in more detail, a time course graph was constructed as shown in Figure 5A. The highlighted areas represent time periods where statistically significant differences between the vibration intensity conditions were observed. The differences among the three vibration intensity conditions are determined based on the average of the alpha frequency band for each highlighted region. The average PSD of the peak period of the alpha ERD (400–600 ms) is significantly stronger for the high- and low-vibration intensity conditions compared to the no vibration condition [one-way ANOVA, *F*_(2, 86)_ = 5.76, Holm–Bonferroni correction, *p_adj*<0.05] as shown in Figure 5B. Note that 2 and 86 in *F*_(2, 86)_ represent 2 degrees of freedom between groups (conditions) and 86 total degrees of freedom, respectively. Furthermore, the average PSD of the ERD rebound (700–2,000 ms) is significantly stronger for the high-vibration intensity compared to low intensity and no vibration conditions [Kruskal–Wallis test, *H*_(2, 86)_ = 19.02, Holm–Bonferroni correction, *p_adj*<0.05], as shown in Figure 5C. Therefore, it is concluded that the ERD in the alpha band in the contralateral somatosensory and motor cortex areas encodes the vibration intensity.

**Figure 6:**
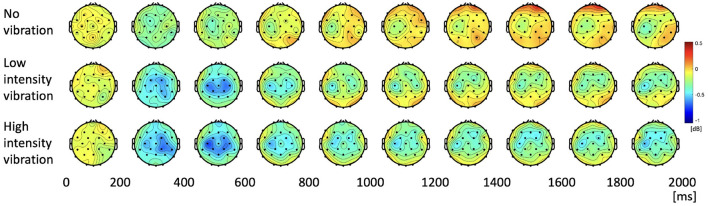
Topographies of beta power spectral density during the task period.

**Figure 7:**
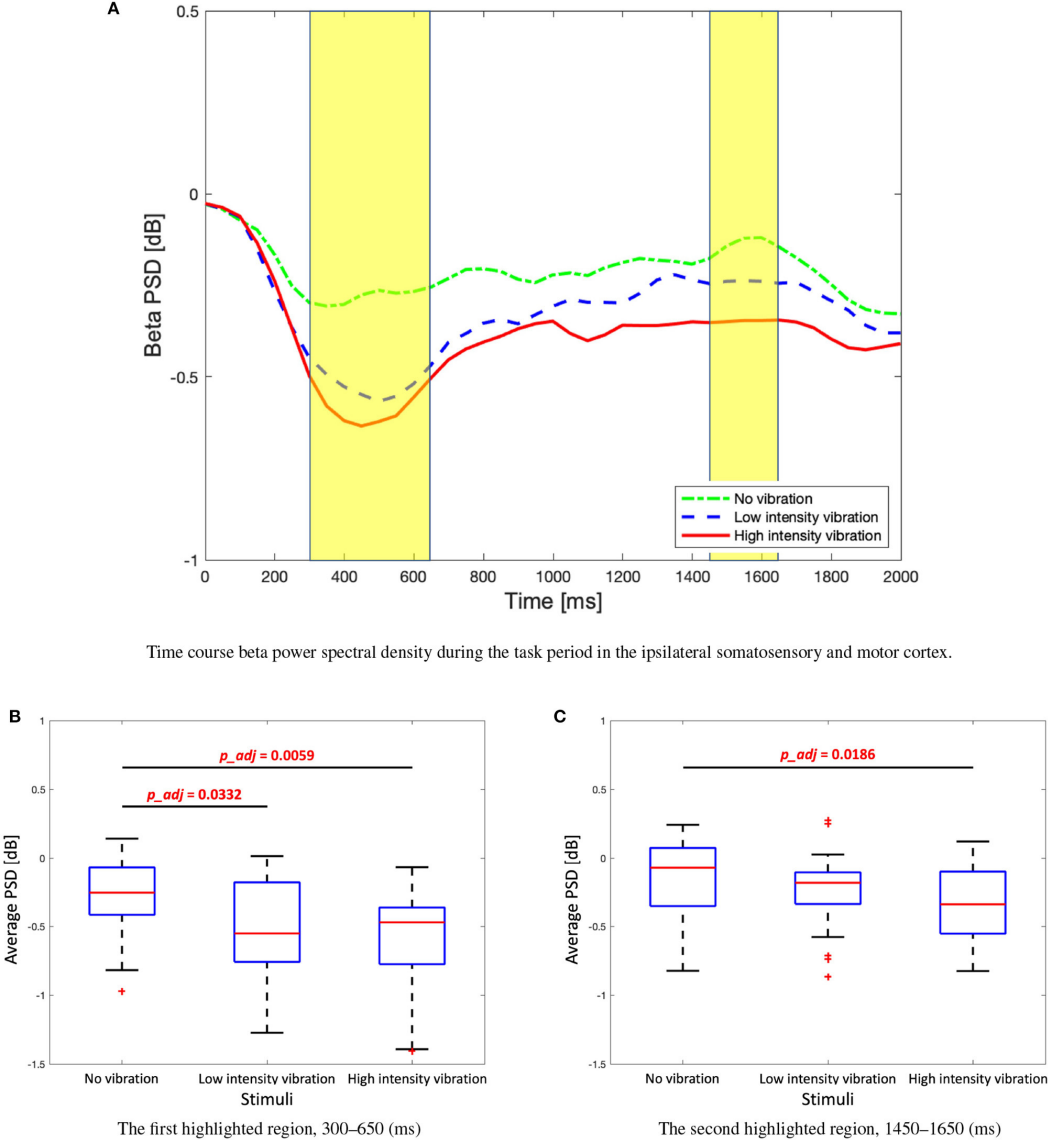
**(A)** Time course beta power spectral density during the task period in the ipsilateral somatosensory and motor cortex (C3, Cp1, and Cp5). Two highlighted periods indicate significant differences among three stimuli. **(B)** Box plots to show average beta power spectral density of the first highlighted regions. One-way analysis of variance (ANOVA), *F*_(2, 86)_ = 6.5, Holm–Bonferroni correction. **(C)** Box plots to show average beta power spectral density of the second highlighted regions. One-way ANOVA, *F*_(2, 86)_ = 4.46, Holm–Bonferroni correction.

The change of the beta PSD shows a slightly different pattern from the change of the alpha PSD. Figure 6 shows that ERD did not appear stronger than that of the alpha band, and rebound was not significant. In the low- and high-vibration intensity conditions, ERDs appeared in the contralateral somatosensory and motor cortices from 200 to 400 ms, and bilaterally from 400 to 600 ms. However, unlike alpha PSD, bilateral ERD did not appear after 600 ms. In addition, Figure 6 shows that ERD was sustained longer in the ipsilateral somatosensory and motor cortices in the beta band, unlike the alpha band, where ERD was sustained longer in the contralateral somatosensory and motor cortices. Therefore, in the beta band, ipsilateral somatosensory and motor cortices (C3, Cp1, and Cp5) were further investigated. The two highlighted sections in the time course beta PSD in Figure 7A were selected by statistical difference among the vibration intensity conditions and are referred to as the peak and rebound periods. In the peak period (300–650 ms), ERD appeared significantly stronger for low- and high-intensity vibration conditions as compared to no vibration condition [one-way ANOVA, *F*_(2, 86)_ = 6.5, Holm–Bonferroni correction, *p_adj*<0.01], as shown in Figure 7B. In the rebound period (1,450–1,650 ms), significant differences remained only between the no vibration and the high-intensity vibration conditions [one-way ANOVA, *F*_(2, 86)_ = 4.46, Holm–Bonferroni correction, *p_adj*<0.05], as shown in Figure 7C. Therefore, beta ERD seems to play a role in encoding the presence of vibration stimulation. The two highlighted sections in Figures 5A, 7A were determined as time windows showing significant differences for the three stimuli.

### 3.3. Event-Related Potential Analysis

**Figure 8:**
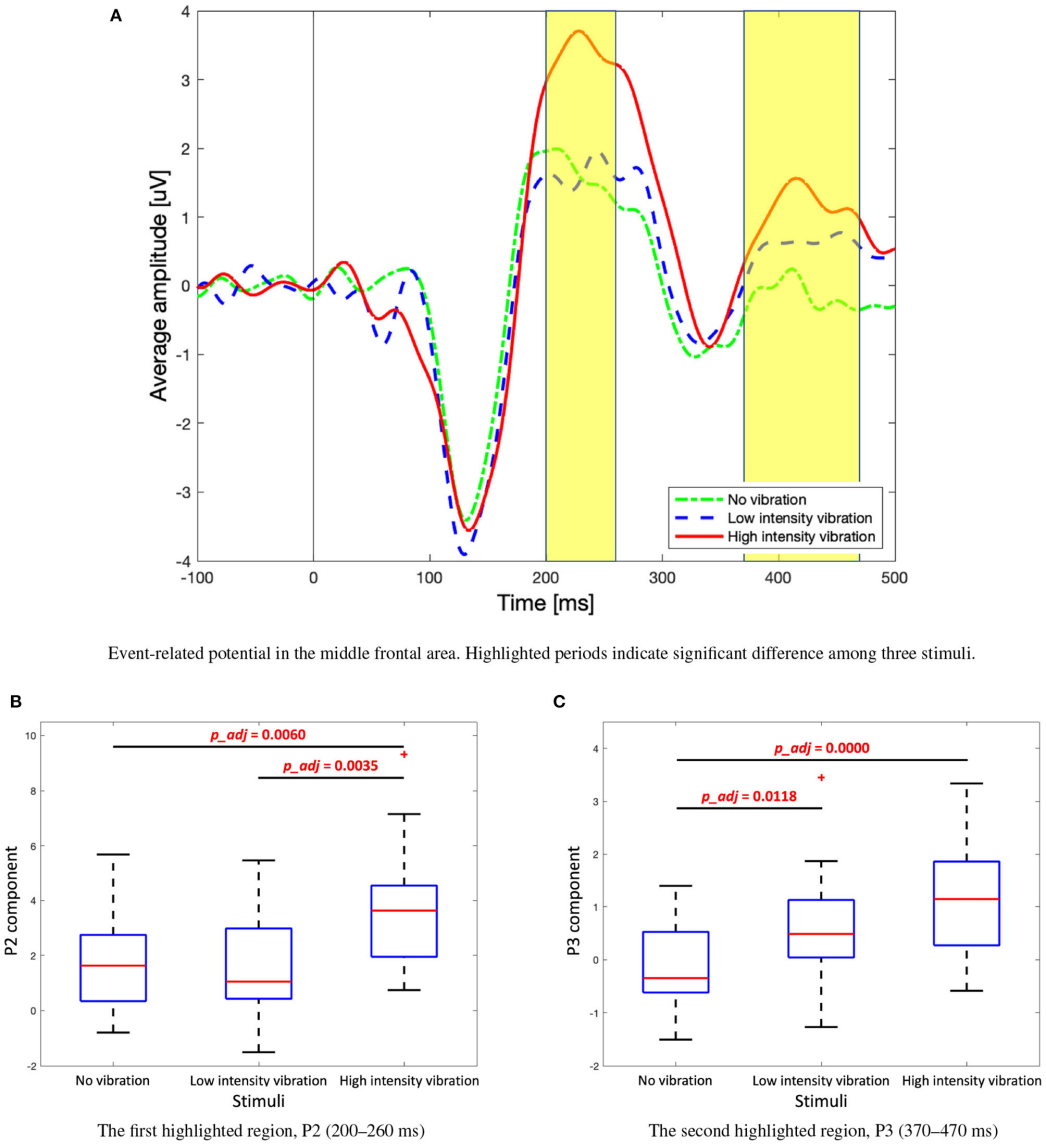
**(A)** Event-related potential, P2, and P3 components in the middle frontal area (Fz). **(B)** Box plots to show significant differences in P2 component among three stimuli. Kruskal–Wallis test, *H*_(2)_ = 14.41, Holm–Bonferroni correction. **(C)** Box plots to show significant differences in P3 component among three stimuli. One-way analysis of variance (ANOVA), *F*_(2, 86)_ = 13.4, Holm–Bonferroni correction.

**Figure 9:**
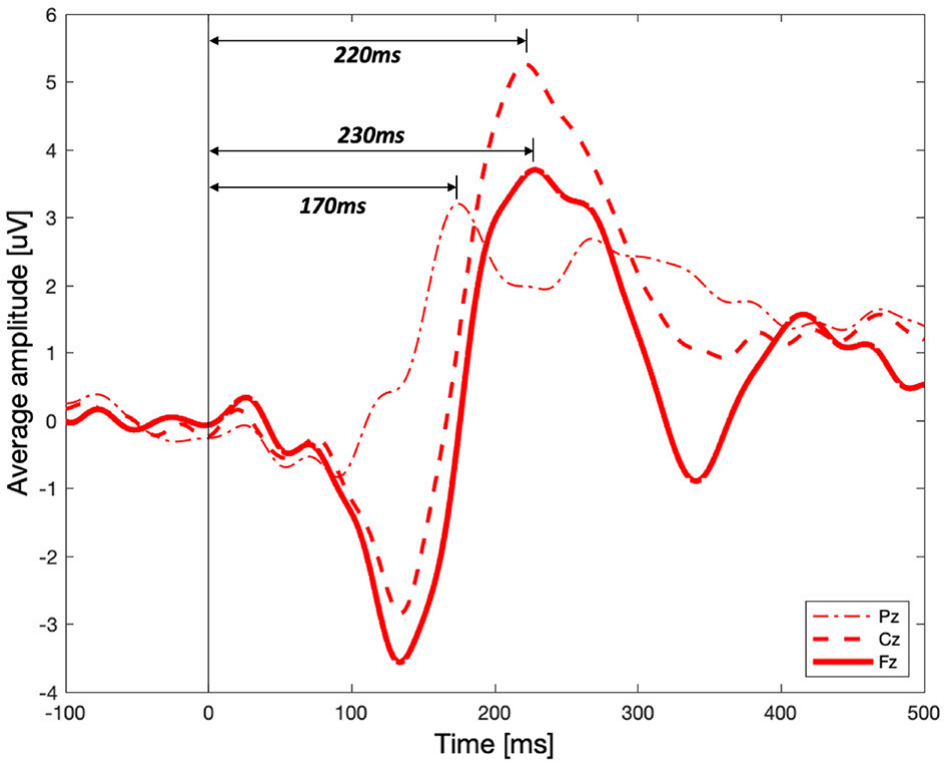
Event-related potential for the high-intensity vibration in the middle parietal (Pz), central (Cz), and frontal (Fz) areas.

Significant differences between the three vibration intensity conditions were observed in the P2 and P3 components of the ERP signal. Figure 8A shows the average amplitude of the three difference vibration intensity conditions in the middle frontal area, Fz, where clear differences between the three conditions are observed. The highlighted periods (200–260 and 370–470 [ms]) indicate time window of P2 and P3, respectively. The P2 component was significantly higher for high-intensity vibration compared to no vibration and low-intensity vibration [Figure 8B, Kruskal–Wallis test, *H*_(2)_ = 14.41, Holm–Bonferroni correction, *p_adj*<0.01]. Furthermore, the P3 component did not appear significantly in the no vibration condition and appeared only in the low-intensity vibration condition [one-sample *t*-test, *t*_(28)_ = 3.38, *p*=0.0021] and high-intensity vibration condition [one-sample *t*-test, *t*_(28)_ = 6.05, *p*=0.0000]. Furthermore, the P3 component for the low- and high-intensity vibration conditions were significantly higher than the no vibration condition [one-way ANOVA, *F*_(2, 86)_ = 13.4, Holm–Bonferroni correction, *p_adj*<0.05], as shown in Figure 8C. The differences of ERP with respect to vibration intensities in each Pz and Cz area were not significant. These results confirm sensory (P2 component) and cognitive (P3 component) processes associated with vibration but do not seem to encode the vibration intensity. In addition to the middle frontal area (Fz), the P2 component was also examined in the middle central (Cz) and middle parietal (Pz) areas. As shown in Figure 9, the peak of the P2 component appeared at 170, 220, and 230 [ms] after the stimulation onset in Pz, Cz, and Fz, respectively. It was found that P2 occurred first in the middle parietal area and subsequently occurred in the middle central area and eventually in the middle frontal area.

## 4. Discussion

### 4.1. Alpha and Beta PSDs

Alpha and beta PSD changes have been reported in many studies related to proprioception or cutaneous sensation (Pfurtscheller et al., 1997; Yao et al., 2017; Angelini et al., 2018; Alsuradi et al., 2020). In this study, changes in alpha and beta PSDs were observed differently. Alpha PSD immediately increased at the onset of the stimulation. Although the participants did not experience any vibration in the no vibration condition, the alpha power increased within 200 ms, especially in the middle central and frontal areas. Therefore, it is reasonable to assume that the increase in alpha power is associated with attention rather than vibration stimulation. Previous research showed that alpha ERD encodes attention (Kerr et al., 2013). The randomize rest time is used to avoid expectation, however it is thought that the participants expected to receive the stimulus soon. Also, due to the rating task for intensity after stimulation period, it is expected that they may concentrate on the short stimulation for two seconds. However, this is not the main concern of this study as it appears the same in all three intensity vibrations.

Alpha ERD activation is driven by stimulation and appeared differently depending on the vibration intensity. Alpha ERD appeared in the no vibration condition, though the participants did not get any vibration stimulation. A previous study showed that alpha/beta ERD appears differently depending on the task (Klostermann et al., 2007), but it is an interesting finding in this study that ERD appeared even with no vibration. Perhaps the participants anticipated that there was vibration stimulation by the task cue, or it might be due to slight finger movements. It would be interesting to use finger tracking techniques (muscle activities using EMG or computer vision) to confirm if ERD activation is indeed due to finger movements. Therefore, additional research is needed to verify these hypotheses. On the other hand, beta ERD was significant for low- and high-intensity vibration but not in the case of no vibration. Even in the case of low- and high-intensity vibration, beta ERD was smaller than alpha, and its rebound was not significant. In addition to this, in Figure 7A, beta rebound did not appear. In general, beta rebound appears after the motor task (Jurkiewicz et al., 2006), however in this experiment, it is thought that it is because the vibration stimuli were given to the fingertips without motor movement, and this is a different result from the alpha PSD.

### 4.2. Strong and Longer ERD Activation in the Time Course Alpha PSD

When participants experienced strong vibration, there are two potential reasons for having a strong and elongated ERD activation. First, strong vibration produces a strong ERD and maintains it for a longer period due to top-down processing. We infer that a stronger ERD occurred due to the human instinct to be more attentive in order to protect oneself against a strong stimulus, and that rebound may be slow because of the prolonged attention. Existing literature also shows that alpha and beta power can be associated with somatosensory attention and top-down cognitive function (Jones et al., 2009; Park et al., 2014).

Another interpretation is that strong vibration may stimulate the mechanoreceptors of the skin more strongly. Thus, the firing of the nervous system may be stronger and last longer. In a study that observed the response of a single neuron by various frequency and amplitude of the vibration stimulation, it was found that for low-intensity stimulation, the frequency of firing of the nerve is small and occurs in a short time, but the frequency of firing is higher and lasts longer as the intensity of stimulation increases (Strzalkowski et al., 2017). The beta rebound after motor movement is reported in many studies (Pfurtscheller et al., 2005; Jurkiewicz et al., 2006), but the alpha rebound is not a common feature. Just few studies show alpha rebound after motor task (Lindig-León et al., 2015). The novelty of this study is to show that the ERD peak and rebound of alpha PSD can be important features in classifying vibration intensity.

### 4.3. Sensation-Related Perception

The P2 component of the ERP was significantly higher in the case of high-intensity vibration compared to the no vibration and low-intensity vibration conditions. This may be attributed to the perceptual processes associated with making a decision about the intensity of the vibration (no vibration and low-intensity vibration conditions were much easier to identify). Through behavioral data, there was a dominant response in the case of no vibration and low-intensity vibration conditions, but the responses for high-intensity vibration were divided comparably between strong and very strong vibration ratings. Furthermore, more than 10% of response for the high vibration intensity was rated as weak vibration. It is concluded that the high-intensity vibration was not as clear for participants to classify as a strong vibration. Choi et al. (2020) shows the difference of SEP for various frequencies and intensities. It shows that SEP peak is increased for strong stimulation, which is similar to the high P2 peak of high-intensity vibration in the ERP result of this study. However, a similar result is also reported by auditory intensity studies (Linka et al., 2005; Paiva et al., 2016).

The P3 component of the ERP also showed differences among the three vibration intensity levels, which is thought to be related to the cognitive efforts for rating the intensity of vibration after completing the task. It is inferred that the cognitive processes involved in rating the high-intensity vibration caused a larger P3. The average 42.06% of the responses for high-intensity vibration were very strong, with a standard deviation of 23.42. On the other hand, the no vibration condition was clearly identified and thus it is assumed that there was no P3 component (average response for no vibration was 99.03%, with a standard deviation of 3.26).

Figure 9 shows that the P2 component for high-intensity vibration shows different latency and amplitude in the middle parietal, central, and frontal areas. Although this result does not show the results according to the three vibration intensities, it helps to understand the tactile sensation that is not yet fully understood. First, it can be seen that the P2 peak occurs in a temporal sequence from parietal to central to frontal areas. It seems that vibration sensation is similar to the dorsal stream transmitted to the frontal area through neural processing with other modalities in the parietal area after basic information is analyzed in the somatosensory cortex. It is well-known that visual information for movement control follows the dorsal stream process, but it has been reported through fMRI studies that tactile and kinesthetic information also follow a similar process (Fiehler et al., 2008). In terms of amplitude, the peak of P2 component in the middle central area was the highest. In the brain–computer interface spellers, P3 of the middle central area (Cz) is shown to distinguish target and non-target stimuli (van der Waal et al., 2012). Middle central area is known to be an important area affecting not only P3 but also P2 for tactile sensation.

This study was intended to investigate the neural representation of vibration intensity, but visual and auditory stimuli were provided in addition to the tactile stimuli for a proper experimental design. A limitation of this study is that, although visual and auditory stimuli were equally provided for no, low, and high vibration intensity conditions, it is an experiment under multimodal stimulation and not only tactile stimulation.

## 5. Conclusion

In this study, we investigated how the vibration intensity is represented in the brain. The time course alpha and beta PSD analysis showed significant differences in ERD associated with the three levels of vibration intensity. Low- and high-intensity vibrations are associated with stronger alpha and beta ERD than no vibration condition. In alpha PSD, rebound of no vibration and low vibration conditions occurred after 700 ms, but in the high-intensity vibration condition, PSD was sustained longer. In addition, the P2 and P3 components of the ERP signal were examined. High-intensity vibration elicited significantly larger amplitude of the ERP, P2 component compared to no vibration and low-intensity vibration. Findings of the present study can be used to provide a quantitative measurement for the perceived vibration intensity based on brain activation.

## Data Availability Statement

The raw data supporting the conclusions of this article will be made available by the authors, without undue reservation.

## Ethics Statement

The studies involving human participants were reviewed and approved by New York University Abu Dhabi and Ulsan National Institute of Science and Technology. The participants provided their written informed consent to participate in this study.

## Author Contributions

ME proposed the study. WP designed the experimental protocol and performed statistical analysis of recorded EEG data. S-PK and ME supervised the study. All authors have contributed intellectually in writing and revising the manuscript.

## Conflict of Interest

The authors declare that the research was conducted in the absence of any commercial or financial relationships that could be construed as a potential conflict of interest.

## Publisher's Note

All claims expressed in this article are solely those of the authors and do not necessarily represent those of their affiliated organizations, or those of the publisher, the editors and the reviewers. Any product that may be evaluated in this article, or claim that may be made by its manufacturer, is not guaranteed or endorsed by the publisher.
